# Isolation of Shiga toxin-producing *Escherichia coli* harboring variant Shiga toxin genes from seafood

**DOI:** 10.14202/vetworld.2018.379-385

**Published:** 2017-03-28

**Authors:** Sreepriya Prakasan, Parmanand Prabhakar, Manjusha Lekshmi, Sanath Kumar, Binaya Bhusan Nayak

**Affiliations:** Department of Post-Harvest Technology, Quality Control Laboratory, ICAR-Central Institute of Fisheries Education, Versova, Mumbai, Maharashtra, India

**Keywords:** *Escherichia coli*, pathogen, seafood, Shiga toxin, Shiga toxin-producing *Escherichia coli*, virulence gene

## Abstract

**Background and Aim:**

Shiga toxin-producing *Escherichia coli* (STEC) are important pathogens of global significance. STEC are responsible for numerous food-borne outbreaks worldwide and their presence in food is a potential health hazard. The objective of the present study was to determine the incidence of STEC in fresh seafood in Mumbai, India, and to characterize STEC with respect to their virulence determinants.

**Materials and Methods:**

A total of 368 *E. coli* were isolated from 39 fresh seafood samples (18 finfish and 21 shellfish) using culture-based methods. The isolates were screened by polymerase chain reaction (PCR) for the genes commonly associated with STEC. The variant Shiga toxin genes were confirmed by Southern blotting and hybridization followed by DNA sequencing.

**Results:**

One or more Shiga toxins genes were detected in 61 isolates. Of 39 samples analyzed, 10 (25.64%) samples harbored STEC. Other virulence genes, namely, *eaeA* (coding for an intimin) and *hlyA* (hemolysin A) were detected in 43 and 15 seafood isolates, respectively. The variant *stx1* genes from 6 isolates were sequenced, five of which were found to be *stx1d* variants, while one sequence varied considerably from known *stx1* sequences. Southern hybridization and DNA sequence analysis suggested putative Shiga toxin variant genes (*stx2*) in at least 3 other isolates.

**Conclusion:**

The results of this study showed the occurrence of STEC in seafood harboring one or more Shiga toxin genes. The detection of STEC by PCR may be hampered due to the presence of variant genes such as the *stx1d* in STEC. This is the first report of *stx1d* gene in STEC isolated from Indian seafood.

## Introduction

*Escherichia coli* have historically been considered as an indicator of fecal contamination of water and food. More than 700 serotypes of *E. coli* have been identified and majority of these are non-pathogenic commensals of the human and animal gastrointestinal tract [1]. However, a small percentage of *E. coli* serotypes have acquired the ability to cause versatile infections ranging from wound infections to fatal meningitis in humans of all age groups [2]. The pathogenic *E. coli* represent a small group of highly specialized strains or serotypes which have specifically evolved to infect humans and animals [3].

*E. coli* causing intestinal infections belong to at least 5 groups of which the Shiga toxin-producing *E. coli* (STEC), also known as the enterohemorrhagic *E. coli* (EHEC), are the most serious pathogens. In the United States alone, STEC infections result in 100,000 illnesses, 3000 hospitalizations and 90 deaths annually [4]. Since animals such as cattle, sheep, and goat are the main reservoirs of STEC, infections generally occur through consumption of meat and milk that are inadequately pasteurized. The primary virulence factor of STEC is the production of one or more Shiga toxins. Shiga toxins (Stx) are of two types; Stx1, which is more or less identical to the toxins produced by *Shigella dysenteriae* 1, and Stx2, which has about 60% similarity with Stx1 [5,6]. Production of one or more Shiga toxins is essential to cause disease, but the production of Stx2 is more correlated with the severity of the disease such as hemolytic uremic syndrome (HUS) and HC [7]. Stx1 is more conserved compared to Stx2 with few variants such as Stx1c and Stx1d, while several sequence variants of Stx2 Stx2a, Stx2b, Stx2c, Stx2d, Stx2e, Stx2f, and Stx2g have been reported [8]. Among these, Stx2 subtypes Stx2a, Stx2c, and Stx2d are commonly associated with severe forms of STEC infections leading to HUS and hemorrhagic colitis (HC) [9,10]. In addition to the production of one more Shiga toxins, several accessory virulence factors are known to determine the pathogenicity of STEC in human infections. Some of the important virulence factors of STEC include the ability to form attachment and effacement lesions (A/E lesions) and production of an enterohemolysin (HlyA) [11]. Although the role of these virulence factors is not fully established, they have been frequently detected if not always, among STEC isolated from clinical cases of HUS and HC. Although roughly 400 serotypes of STEC produce Shiga toxins, all of them are not implicated in human infections. Serotype O157:H7 is the most common serotype involved in severe cases of infections leading to HUS and HC, and majority of the large-scale outbreaks of STEC infections have been associated with this serotype [12]. Several other serotypes, generally termed as non-O157 serotypes such as O26, O45, O91, O103, O111, O121, and O145, are also involved in severe human infections [13,14].

Since food animals harbor STEC, foods of animal origin are naturally contaminated with STEC [15]. Fecal shedding of STEC and subsequent contamination of surrounding environments leads to spread of STEC to other foods. The occurrence of STEC in seafood is due to secondary contamination from water, handlers of seafood or cross-contamination from other foods [16-18]. STEC isolated from seafood in India, by far, are of non-O157 serotype [16,18]. The true pathogenic potentials of these isolates are not known. Since seafood is contaminated from terrestrial sources, it is expected that STEC isolates from seafood could be serologically and genetically very diverse. Further, aquatic environment is an ideal niche for complex genetic interactions involving exchange of genetic materials among closely related bacteria leading to the evolution of new pathogens.

In the present study, STEC were isolated from seafood and characterized for the commonly associated virulence genes. The presence of Shiga toxin genes was tested using different sets of primers, and the putative variant *stx* genes were sequenced. The sequence variants were compared to identify the occurrence of a new variant *stx* gene in seafood isolates of STEC.

## Materials and Methods

### Ethical approval

Since no animals were used in this study, ethical approval was not needed.

### Collection of samples and bacteriological analysis

A total of 39 seafood samples (18 finfish and 21 shellfish) were collected during August 2014-April 2015 from landing centers and retail fish markets in the western Mumbai, India. The samples were placed in a sterile plastic bag and transported to the laboratory in chilled condition within 1 h of collection for bacteriological analysis. Tryptone phosphate broth (TP) and HiCrome EC broth (HiMedia, Mumbai) were used as selective enrichment broths [19]. 25 g of the sample was aseptically weighed and transferred to a stomacher bag (Seward Medical, London, UK), homogenized for 60 s in a stomacher (Seward Stomacher 80, Lab system, London, UK) and added to 225 mL of TP broth or HiCrome EC broth. The TP broth was incubated at 44.5°C for 24 h, while HiCrome EC broth was incubated at 37°C for 24 h. Following incubation, two loopful from the enrichment broths were streaked on two different selective agar plates Sorbitol MacConkey Cefixime Agar (SMAC) (HiMedia, Mumbai) supplemented with tellurite-cefixime and HiCrome O157:H7 agar (HiMedia, Mumbai) supplemented with potassium tellurite. The plates were incubated for 18-24 h at 37°C. Typical *E. coli* colonies, 3-5 from each selective plate, were purified on Luria Bertani Agar plates and subjected to biochemical tests for the identification for *E. coli* which included oxidase test, indole production, methyl red-Voges–Proskauer, and citrate utilization tests [19].

### Extraction of genomic DNA

Genomic DNA from *E. coli* isolates was extracted using cetyltrimethylammonium bromide (CTAB) method [20]. Briefly, a 5 mL of the culture was centrifuged, and the pellet was resuspended in 456 µL of 1× TE (1 mM Tris, pH 8; 10 mM EDTA) buffer, followed by the addition of 30 µL of 10% SDS and 3 µL of proteinase K (20 mg/mL). The mixture was incubated for 1 h at 37°C. Following this, 100 µL of 5% CTAB and 80 µL of 100 mM NaCl were incubated at 65°C for 15 min. The mixture was extracted with an equal volume (600 µL) of phenol, chloroform, and isoamyl alcohol (25:24:1) and centrifuged at 10,000 rpm for 10 min. The aqueous layer was extracted with an equal volume chloroform and isoamyl alcohol (24:1) and centrifuged at 10,000 rpm for 10 min. DNA was precipitated using 0.6 volumes of isopropanol, washed with 70% ethanol, air dried and dissolved in 1× TE buffer. The concentration and the purity of DNA were measured using Nanodrop spectrophotometer (Thermo Scientific, USA).

### Polymerase chain reaction (PCR) for virulence genes of STEC

The *E. coli* isolates were subjected to PCR for the detection of virulence genes commonly associated with STEC using previously described oligonucleotide primers and thermocycling conditions (Table-1) [21-27]. All PCRs were performed in a Prima-96 thermocycler (HiMedia, Mumbai). The amplicons were separated on a 1.6% agarose gel, stained with ethidium bromide (HiMedia, Mumbai) and photographed using a gel documentation system (Bio-Rad, USA). An EHEC strain EDL 933 was used as the positive control in all PCR reactions.

**Table-1:** PCR primers used in this study and their target genes.

Primer	Target gene	Sequence (5’-3’)	Product size (bp)	Reference
stx1F-PA	*st*x*1*	ATAAATCGCCATTCGTTGACTAC	180	[23]
stx1R-PA		AGAACGCCCACTGAGATCATC		
stx2F	*st*x*2*	GGCACTGTCTGAAACTGCTCC	255	
stx2R		TCGCCAGTTATCTGACATTCTG		
eaeAF	*eaeA*	GACCCGGCACAAGCATAAGC	384	
eaeAR		CCACCTGCAGCAACAAGAGG		
hlyAF	*hlyA*	GCATCATCAAGCGTACGTTCC	534	
hlyAR		AATGAGCCAAGCTGGTTAAGCT		
VT-comF	Common for *st*x*1* and *st*x*2*	GAGCGAAATAATTTATATGTG	518	[21]
VT-comR		TGATGATGGCAATTCAGTAT		
stx1F-FA	*st×1*	CCATGACAACGGACAGCAGTT	779	[22]
stx1R-FA		CCTGTCAACTGAGCAGCACTTTG		
stx2F-FA	*st*x*2*	GTGGCGAATACTGGCGAGACT	890	
stx2R-FA		CCCCATTCTTTTTCACCGTCG		
eaeAF-FA	*eaeA*	ACACTGGATGATCTCAGTGG	614	
eaeAR-FA		CTGAATCCCCCTCCATTATG		
hlyAF-FA	*hlyA*	ACGATGTGGTTTATTCTGGA	166	
hlyAR-FA		CTTCACGTGACCATACATAT		
st×1C-F	*st*x*1c* variant	TTTTCACATGTTACCTTTCCT	498	[26]
st×1C-R		CATAGAAGGAAACTCATTAG		
MK1	Common for *st*x*1* and *st*x*2*	TTTACGATAGACTTCTC*GA*C	230	[24]
MK2		CACATATAAATTATTTCG*C*TC		
BGRIU	*st*x*1*-full	TCAACGAAAAATAACTTCGCTGAATCCC	1178	[25]
BGRID		CAGTTAATGTGGTTGCGAAGGAATTTACC		
BGR2U	*st*x*2* -full	ATGAAGTGTATATTATTTAAATGGGTACTGTG	1226	
BGR2D		TCAGTCATTATTAAACTGCACTTCAG		
VT1AF	*st*x*1*-full	TCGTATGGTGCTCAAGGAGT	966	[27]
VT1AR		AGTTCTGCGCATCAGAATTG	1309	
VT1BR2		AGAACCGGCAACAACTGACT		

Thermocycling conditions: 94°C-1 min, 55°C-1 min, 72°C-1 min for *st×1*, *st×2*, *hlyA*, *eaeA*, and *st×1* full gene amplification (BGRIU/BGRID); 94°C-1 min, 51°C-1 min, 72°C-1 min for *st×1c*; 94°C-1 min, 55°C-1 min, 72°C-2 min for *st×2*-full gene amplification (BGR2U/BGR2D and VT1AF/VT1AR/VT1BR2), PCR=Polymerase chain reaction

### Southern blotting and hybridization

The non-specific *stx2* amplicons obtained with 12 *E. coli* isolates were subjected to Southern hybridization to determine if they contained Shiga toxin gene sequences. A biotin-labeled probe was prepared using *stx2* PCR product from the reference strain USFDA (serotype O157:H7) using Biotin DecaLabel DNA labeling kit (Thermo Scientific, USA). PCR products were separated on a 1.5% agarose gel and Southern blotted onto a positively charged nylon membrane (SensiBlot Plus, Thermo Scientific, USA) in an alkaline condition [28], and hybridized with the biotin-labeled probe at 42°C overnight [29]. Following this, the membrane was washed twice with 5× SSC, 0.5% [W/V] SDS at 50°C for 5 min each, 0.1× SSC, 1% [W/V] SDS at 42°C for 15 min each, and once with 2× SSC for 5 min at room temperature. The membrane was incubated with a blocking solution for 1 h at 60°C, followed by incubation with streptavidin-alkaline phosphatase (1:5000 diluted) for 10 min at room temperature. The color development was done by placing the membrane in BCIP-NBT (330 µg/mL NBT and 167 µg/mL BCIP) solution in the dark with gentle shaking. The color reaction is terminated by rinsing the membrane in stop solution (20 mM Tris-HCl pH 7.5, 5 mM EDTA).

### Nucleotide sequence accession numbers

The nucleotide sequences of *stx1* genes derived in this study have been deposited in GenBank under accession numbers KR632986, KR632985, KR632984, KR632983, and KR632982.

## Results

### Isolation of STEC from seafood samples

Of 670 isolates screened from 39 samples finfish and shellfish, 368 (54.9%) were confirmed as *E. coli* isolates by biochemical tests and PCR. A total of 10 (25.64%) seafood samples were positive for STEC which comprised 3 fish and 7 shellfish samples (Table-2). *E. coli* were present in all the samples (100%) analyzed in this study. When the numbers of isolates from different selective agars were compared, of 368 isolates from seafood samples, 258 (70%) were from SMAC, and 110 (30%) were from HiCrome agar suggesting that SMAC allowed better isolation of *E. coli* from seafood compared to the chromogenic agar. Further, of 368 *E. coli* isolates 208 (56.5%) were from fish and 160 (43.5%) were from shellfish.

**Table-2:** Details of seafood sample analyzed and the isolation of STEC.

Type of sample	Number of samples (n)	Number (%) of STEC positive samples	Number of STEC* isolated
Fish	18	3 (16.6)	25
Shellfish	21	7 (33.33)	36
Total	39	10 (25.64)	61

* Positive for at least one *stx* gene, STEC=Shiga toxin-producing *Escherichia coli*

#### Genotyping of STEC isolates

**Figure-1:**
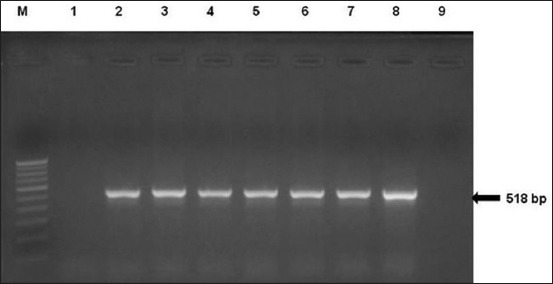
Amplification of *stx* gene using a common primer for both *stx1* and *stx2* genes. Lane M: 100 bp DNA ladder, Lanes 2-7: *Escherichia coli* isolates from oysters and clams, Lane 8: Positive control (*E. coli* O157:H7), Lanes 1 and 9: Negative control.

The common primers VTcom-F and VTcom-R [21] amplifying common regions of *stx1* and *stx2* genes detected Shiga toxin genes in 61 isolates. Further, seven STEC isolates which included 4 isolates from clams (CEC-1, CEC-2, CEC-3, and CEC-4) and 3 from oysters (OYEC-5, OYEC-6, and OYEC-7) were among those which yielded specific amplicons with common primers (Table-1) [21-29], but not with specific *stx1* or *stx2* primers (Figure-1). These were further characterized by sequencing the full length *stx* gene amplified using primers BGRIU and BGRID (Table-1) [21-29]. Sequence analysis of 1.3 kb *stx* genes from 5 isolates revealed that these isolates are positive for *stx1* gene of *stx1d* variant type. The seven *stx1*-positive isolates were further characterized for additional virulence genes by PCR and were found to be negative for *stx2* gene, *hlyA*, and *eaeA* genes. Further, the *stx* gene of isolate CEC-1 could not be amplified using stx common primers, as well as the *stx1*-specific primers [22], but could be amplified using another set of *stx1*-specific primers [23]. Similar discrepancies with *stx* amplifications could be seen with the isolate CEC-5, presumably due to sequence differences in primer binding sites. The full sequence of *stx1* gene in 5 isolates (CEC-2, CEC-3, CEC-4, OYEC-5, and OYEC-6) was obtained using different combination of primers stx1F-PA and BGRID (Table-1) [21-29].

**Figure-2:**
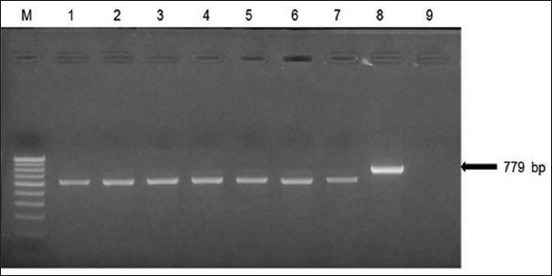
Polymerase chain reaction (PCR) amplification of *stx2* genes in *Escherichia coli* isolates from fish using primers stx2F-FA and *stx2*R-FA. The isolates yielded smaller amplifications products compared to the expected product of 779 bp. The presence of *stx2* sequences in the amplicons was confirmed by sequencing of PCR products. Lane M: 100 bp DNA ladder, Lanes 1-7: *E. coli* isolates from *Bregmaceros mcclellandi* (spotted codlet), Lane 8: Positive control (*E. coli* O157:H7), lane 9, negative control.

**Figure-3:**
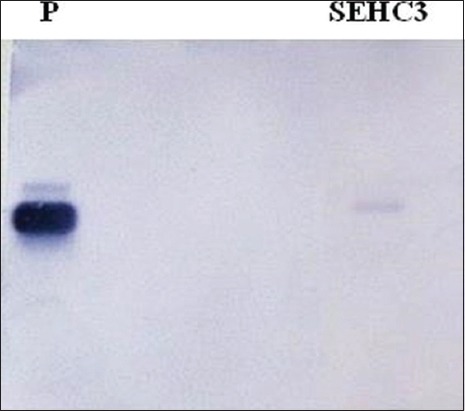
*Escherichia coli* SEHC3 isolated from the fish *Scomberomorus commerson* yielded faint amplicon with a *stx2*-specific polymerase chain reaction (PCR). The presence of *stx2* gene sequence in the amplicon was confirmed by Southern hybridization with a biotin-labeled probe. Lanes P: *stx2* amplification product of reference strain *E. coli* O157:H7, Lane SEHC3: *stx2* PCR product of SEHC3.

A few *E. coli* isolates, 22 in number, which yielded weak amplicons with common *stx* primers [21], did not yield any amplification with either *stx1*- or *stx2*-specific primers (data not shown). It was hypothesized that isolates could be harboring sequence variants of Shiga toxin genes which were not amplifiable by existing primers [16]. These isolates were screened for *stx* genes using a different pair of *stx* common primers, MK1 and MK2 (Table-1) [21-29]. Four isolates showed expected amplicons, while the remaining isolates yielded non-specific amplicons (data not shown). Further, 7 isolates (SST1, SST2, SST3, SST4, SST5, SST6, and SST7) from a sample of fish showed amplicons of different but uniform sizes with *stx2*-specific primers (Figure-2). The PCR products obtained with SST1-SST7 were sequenced and BLAST analysis revealed that these were indeed *stx2* gene amplification products. All 7 isolates were positive for *hlyA* and *eaeA* genes confirming their identity as STEC. In addition, an isolate of *E. coli* (SEHC3) consistently yielded faint amplification with *stx2* primers stx2F-FA and stx2R-FA [22]. Since the sequencing of this faint amplicon was not possible, Southern hybridization was performed using biotin-labeled polynucleotide probe derived from the *stx2* PCR product of O157:H7 reference strain. The *stx2* PCR product of SEHC3 reacted with the probe suggesting the presence of *stx2* sequence (Figure-3).

## Discussion

The presence of *E. coli* in seafood indicates fecal contamination from human-animal sources since *E. coli* are not normal inhabitants of the coastal-marine environments [30]. Since a proportion of *E. coli* can harbor genes that can contribute to their ability to cause a range of infections in humans and animals, the focus on *E. coli* in food has now been shifted from their role as indicators of fecal contamination to important agents of food-borne human infections. The contamination of coastal-marine environments from anthropogenic sources will lead to contamination of seafood harvested from such waters with human enteric pathogens. Routine monitoring of coastal environments and the seafood will help in identification of critical points of contamination of seafood and plan scientific interventions to prevent such contaminations. In this regard, the present study was aimed at screening fresh seafood for the presence of STEC. STEC was detected in 10 (25.64%) seafood samples (Table-2). For the isolation of pathogenic *E. coli*, two enrichment broths (TP broth and HiCrome EC broth) and two selective agars (SMAC agar and HiCrome O157:H7 EC agar) were used. Both the selective agars contained antibiotics as selective agents. Pathogenic strains of *E. coli* occur in very small numbers in a background of large number of other related enterobacteria which may outgrow the pathogenic *E. coli* when less selective media are used. However, these two selective agars varied with respect to the number of *E. coli* isolated on them, with SMAC yielding 70% and HiCrome O157:H7 agar yielding 30% of all the isolates of *E. coli* obtained in this study.

Several studies in the past two decades have reported the prevalence of STEC in different types of foods including seafood in India. The first report on the isolation of STEC from India was from non-diarrheic animal sources [31]. STEC were isolated and characterized from animals, humans and food products, as well as from diarrheic calves and lambs [32,33]. In an elaborate study [34], 876 samples which included 330 animals, 184 humans, and 362 food samples were screened, and 17 STEC strains were isolated. These isolates harbored different combinations of virulence genes such as *stx1* and *stx2* (44.5% of strains), *stx2* alone (44.5%) and *stx2* and *hlyA* (19%) [33]. Studies on the prevalence and characterization of STEC in seafood in India are limited. The occurrence of STEC in seafood in India was first reported in 2001 [18]. In this study, STEC were isolated from 5% of clams and 3% of fresh fish samples [18]. Seafood isolates of STEC harbored different combinations of *stx* genes and were mostly of non-O157 serotypes [16]. A study from Cochin, India reported the isolation of O157:H7 from shrimp samples [35]. These studies strongly suggest the association of STEC with seafood, although the exact sources of these STEC have not been scientifically demonstrated.

In this study, different PCR primers and protocols were used detect Shiga toxin genes of STEC associated with seafood. 61 isolates of *E. coli* harbored one or more Shiga toxin genes. No single primer pair was able to detect *stx* genes in all isolates of this study. Sequencing and Southern hybridization were performed to detect *stx* gene sequences in cases of ambiguity. Nucleotide sequencing enabled detection of *stx2* gene sequences in 7 STEC isolates (SST1-SST7) from a fish sample (Figure-2). The presence of *hlyA* and *eaeA* genes in these isolates further confirmed their identity as STEC. However, amplification of full-length *stx2* gene from these isolates was not successful using primers shown in Table-1 [21-29]. Further, an isolate of *E. coli* (SEHC3) which consistently yielded faint amplification with *stx2* primers stx2F-FA and stx2R-FA [22] was further analyzed to determine if it indeed harbored *stx2* gene by Southern hybridization using biotin-labeled polynucleotide probe. The *stx2* PCR product of SEHC3 reacted with the probe suggesting the presence of *stx2* sequence (Figure-3). This is presumably a variant *stx2* gene and its characterization by cloning and sequencing is currently in progress. Although more than 20 sequence variants of *stx2* have been reported, these are placed under 7 major subtypes designated as *stx2a, stx2b, stx2c, stx2d, stx2e, stx2f*, and *stx2g* [8]. There are not many reports on the occurrence of STEC with variant *stx* genes in seafood from India.

Although the primer combinations BGRID [25] and stx1F-PA [23] amplified whole *stx1* genes in 6 isolates, the combination failed to amplify the gene in CEC-1. However, by replacing the reverse primer stx1F-PA with stx1R-FA [22], the *stx1* gene of CEC-1 could be amplified. This suggests that the isolate CEC-1 could be harboring a variant *stx1* gene not reported so far. All isolates except CEC-1 were positive by *stx1*c PCR [26], but their *stx1* gene sequences were not identical with *stx1c*. Stx1c has 97.1% and 96.6% amino acid sequence identity in its A and B subunits, respectively, with the *stx1* encoded by bacteriophage 933J [26]. Stx1d has a greater sequence variation with only 91% amino acid identity with the Stx1 of 933J [27]. The use of different combinations of *stx* primers and sequencing of full-length *stx1* gene enabled the identification of *stx1d* variants in this study, which otherwise would have been misidentified as *stx1c*.

The presence of variant *stx* genes has several implications. First, the genes may be missed by regular PCR using primers designed based on known sequences [16,27]. Further, STEC harboring variant genes may represent their reservoirs, such as the *stx1* variant Stx1OX3 which is of ovine origin [36]. *stx1c*, for example, has not been reported from EHEC serogroups O26, O103, O111, O145, or O157 and *stx1c*-harboring *E. coli* lack *eae* gene [26]. This also points out at the fact that STEC harboring variant Shiga toxin genes may vary greatly in their virulence gene compositions and hence in their virulence to humans.

Previous studies from India have shown the presence of *stx* gene variants in STEC isolated from different sources. A study reported that *stx1, stx1c, stx2c, stx2d* genes were prevalent in STEC isolates of human origin in India [33]. Of 187 fecal samples tested, *stx1c* was found in 13 (30.70%) isolates, while *stx2c* and *stx2d* were found in 8 (24.24%) and in 2 (6.06%) isolates, respectively [33]. A study conducted to determine the prevalence and distribution of various variants of *stx* gene in STEC isolates from the animal stool, meat, and human illness reported the presence of *stx1d* gene [37]. Kumar et al. reported *stx1c* gene from beef samples of Mangalore, India [16]. These *stx1c*-positive isolates lacked *eaeA* and *hlyA* genes [16]. The present study constitutes the first report on the prevalence of STEC harboring *stx1d* variant genes in seafood from India.

The results of this study proved the occurrence of STEC in fresh seafood and also STEC with variant Shiga toxin genes. The presence of *E. coli* with pathogenic potentials such as the STEC is of great concern. As seen in this study, seafood isolates of STEC are very diverse in contrast to clinical isolates which are more or less homogenous. The presence of diverse genotypes of STEC in seafood may be attributed to diverse sources of contamination of seafood. Since some of the virulence genes such as *stx* and *hlyA* are phage or plasmid encoded, dissemination of virulence genes in the aquatic environment may result in the emergence of more virulent strains of pathogens. Further, based on the PCR and sequencing results of this study, it may be hypothesized that more variants of Shiga toxin genes may exist in the environment in the region of this study. Probe hybridization and sequencing will help to identify the variant Shiga toxin genes in seafood associated STEC.

## Authors’ Contributions

SK conceived and designed the experiments; SP and PP performed the experiments. SK, BBN, and ML collectively planned and supervised the experiments and analyzed the data. All authors read and approved the final manuscript.
